# Supplementary figures and images for: Nuclear receptors NHR-49 and NHR-79 promote peroxisome proliferation to compensate for aldehyde dehydrogenase deficiency in C. elegans
Source: PLoS Genet. 2021 Jul 8;17(7):e1009635. doi: 10.1371/journal.pgen.1009635 (PMC8291716; doi:10.1371/journal.pgen.1009635)

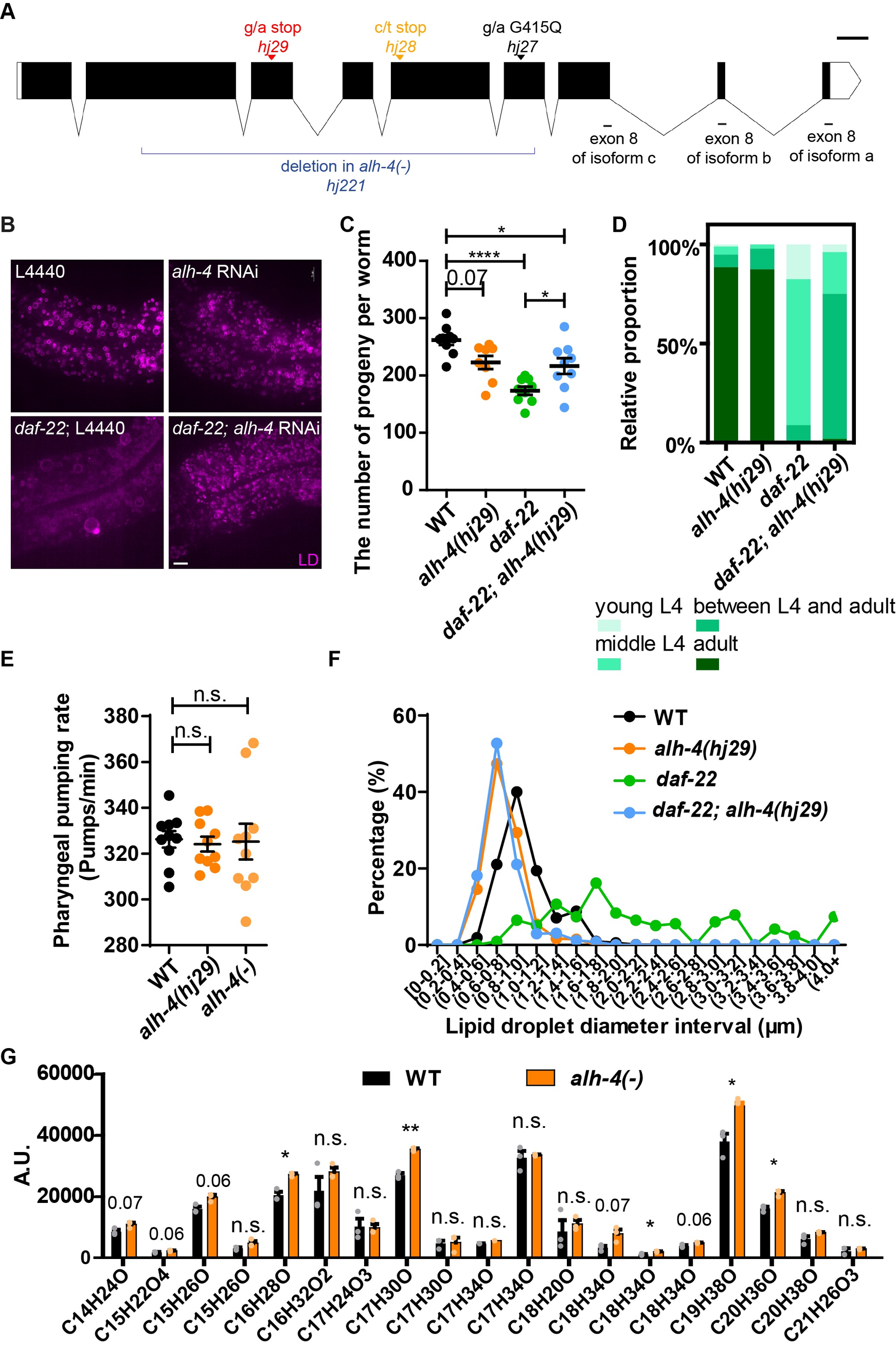

Supplement: S1 Fig — (A) Schematic representation of the alh-4 gene structure. Black boxes, exons; white boxes, untranslated region; line, splicing. The mutation sites in hj28 and hj29 are indicated by red triangle and yellow triangle, respectively. The single nucleotide substitution in both alleles result in a premature stop codon. The mutation site in hj27 is indicated by black triangle and the single nucleotide substitution results in a missense mutation Gly 415 Glu. The deletion region in alh-4 in hj221 is indicated with the blue line. alh-4(hj221) is labeled as alh-4(-) in all figures. Scale bar = 100bp. (B) Representative images of lipid droplets in the second intestinal segment of L4 worms. Lipid droplets were labeled with mRuby::DGAT-2 (hjSi112). Scale bar = 5μm. (C) Quantification of number of progeny of wild type (WT) and mutant worms of indicated genotypes. Two-way ANOVA with Tukey’s multiple comparisons test was applied. (D) Percentage of 50 synchronized worms in the young L4, middle L4, between L4 and adult or adult developmental stage observed at 70 hours after egg laying. (E) Quantification of the pharyngeal pumping rate of WT, alh-4(hj29) and alh-4(hj221) worms. n = 10 for each group. Two-tailed unpaired Student’s t-test was applied. (F) Frequency distribution of LD diameter of wild type (WT) and mutant worms of indicated genotypes. Total number of the lipid droplet measured: WT = 1055, alh-4(hj29) = 1628, daf-22(ok693) = 216, alh-4(hj29); daf-22(ok693) = 943. (G) Quantification of indicated fatty aldehydes and fatty alcohols in wild type (WT) and alh-4(-) worms with LC-MS. A significant increase in multiple species of fatty aldehyde/fatty alcohol was observed in alh-4(-) worms in comparison with WT worms. n = 3 for each group except 0 value was considered as missing value (NA) and therefore ignored. Each dot represents the average value of two technical replicates of each biological sample. Two-tailed unpaired Student’s t-test was applied. For all plots, mean ± SEM of e [file pgen.1009635.s001.tif]

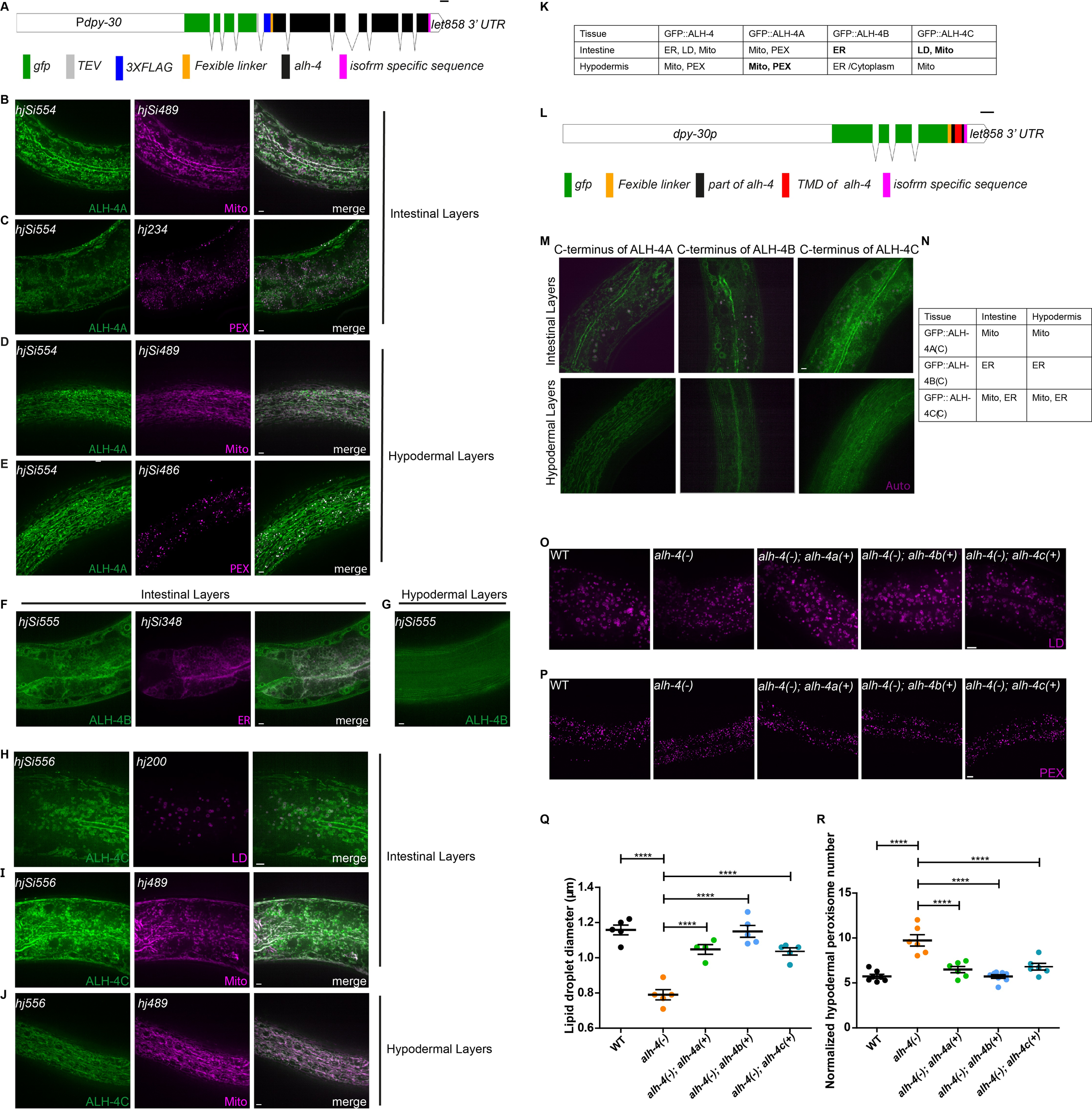

Supplement: S2 Fig — (A) Schematic representation of the structure of transgenes expressing GFP tagged ALH-4 isoforms. The magenta box indicates isoform specific sequence. ALH-4A isoform: AAATATGCTCGCAATCTTCATTGA encoding KYARNLH* ALH-4B isoform: TTCATTTTCCGCTTCTCGGCATAA encoding FIFRFSA*. ALH-4C isoform: GTATGCAGAGGAAAATCAGCTCAATAG encoding VCRGKSAQ*. Scale bar = 100bp. (B—E) GFP::ALH-4A (hjSi554) colocalized with mitochondria labeled with TOMM-20N::mRuby (hjSi489) and peroxisomes labeled with tagRFP::DAF-22 (hj234) in the intestine and tagRFP::PTS1 (hjSi486) in the hypodermis. (F) GFP::ALH-4B (hjSi555) colocalized with the ER labeled with ACS-22::tagRFP (hjSi348) in the intestine. (G) No distinct pattern of GFP::ALH-4B was detected in the hypodermis. (H—I) In the intestine, GFP::ALH-4C (hjSi556) colocalized with lipid droplets labeled with DHS-3::mRuby (hj200) and mitochondria labeled with TOMM-20N::mRuby (hjSi489). (J) In the hypodermis, GFP::ALH-4C colocalized with mitochondria labeled with TOMM-20N::mRuby (hjSi489). (K) Table summarizing the subcellular localization of different ALH-4 isoforms in the intestine and hypodermis. (L) Schematic representation of the structure of transgenes expressing GFP fused with the C-terminus of ALH-4 isoforms. Scale bar = 100bp. (M) Representative images showing the subcellular localization of GFP fused with C-terminus of ALH-4 isoforms in the intestine (top) and in the hypodermis (bottom). (N) Table summarizing the results shown in (M). (O) Transgenic rescue of alh-4(-) worms with specific ALH-4 isoforms. Representative images showing intestinal lipid droplets labeled with DHS-3::mRuby(hj200). (P) As in (O), but with representative images showing the hypodermal peroxisomes labeled with tagRFP::PTS1(hjSi486). (Q) Quantification of intestinal lipid droplet diameter. n = 5 for each group. (R) Quantification the hypodermal peroxisome number normalized with the length of the worm in the field of view. n ≥ 6 for each group. In each plot, mean ± SEM of e [file pgen.1009635.s002.tif]

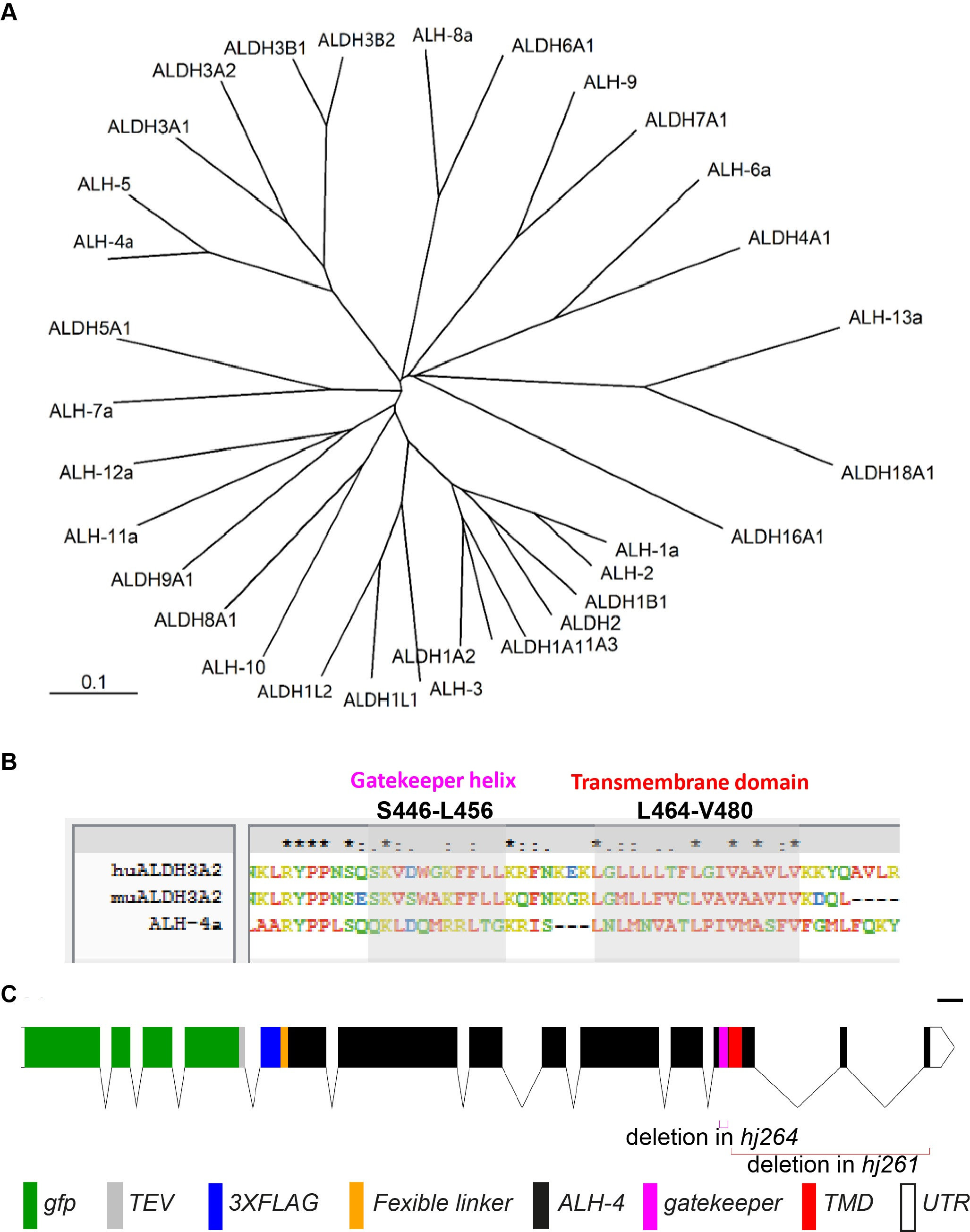

Supplement: S3 Fig — (A) Phylogram of human ALDHs and C. elegans ALHs. ALH-4 is most closely related to class III ALDHs based on their primary amino acid sequences. The length of the branch shows the genetic distance. For human proteins with isoforms, the amino acid sequence of the longest isoform was used for sequence comparison. (B) Sequence alignment of human and mouse ALDH3A2 and C. elegans ALH-4A (with Clustal X2). Residue color scheme: hydrophobic (A, L, M, P, I, F, W, V), red; polar (N, C, Q, T, Y, G, S), green; negative charged (D, E), blue; positive charged (H, R, K), yellow. Alignment quality display: ‘*’ for a single, fully conserved residue; ‘:’ for fully conserved of some “strong groups”; ‘.’ for fully conserved of some “weak groups”; Corresponding regions are in grey areas. (C) Schematic representation of gfp::alh-4(Δgk) (hj264) and gfp::alh-4(ΔC) (hj261), which had the gate-keeper region and C-terminal membrane anchor deleted, respectively. The deleted region is indicated. TMD, transmembrane domain. Scale bar = 100bp. (TIF) [file pgen.1009635.s003.tif]

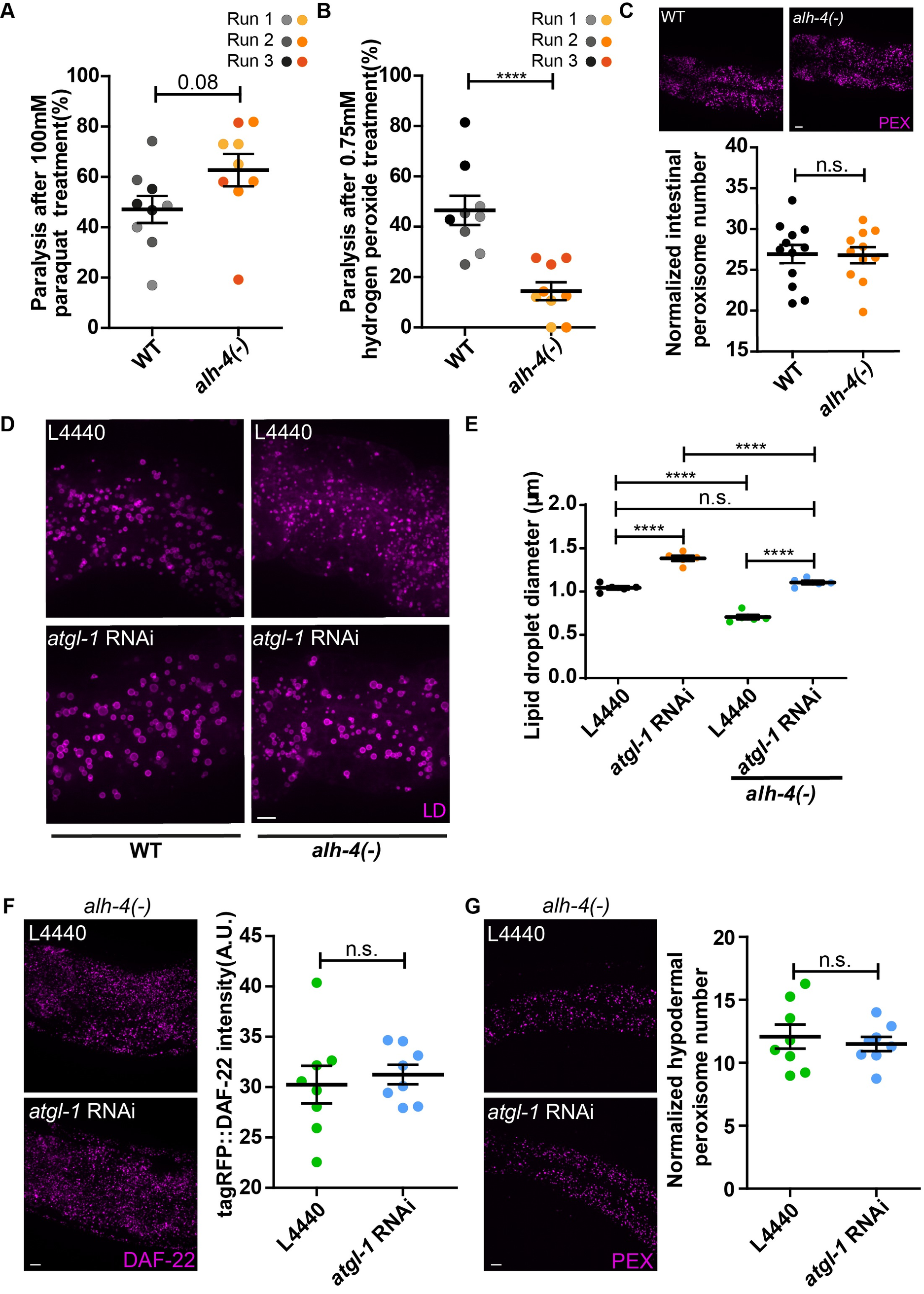

Supplement: S4 Fig — (A) Scatter plot showing the percentage of worms paralyzed after being treated with 100mM Paraquat for 6 hours. Three independent experiments were carried out and each with three biological replicates. The total number of worms measured in 9 biological replicates were 4, 13, 7 for WT and 34, 8,12 for alh-4(-) in the first run; 8, 10, 36 for WT, 19, 12, 13 for alh-4(-) in the second run and 14, 21, 19 for WT and 25, 10, 11 for alh-4(-) in the third run. Data points are color-coded to indicate results from specific experiments. (B) As in (A) but showing the percentage of worms paralyzed after 4 hours treatment with 0.75mM hydrogen peroxide in M9 buffer. The total number of worms measured in 9 biological replicates were 41, 27, 35 for WT and 32, 28, 33 for alh-4(-) in the first run; 16, 21, 22 for WT and 17, 24, 35 for alh-4(-) in the second run and 25, 28, 27 for WT and 29, 28, 29 for alh-4(-) in the third run. (C) Representative images (Z-stack, 0.5 μm intervals, 10 slices) showing the intestinal peroxisomes labeled with mRuby::PTS1 (hjSi548) (top). Quantification of intestinal peroxisome number (bottom). n = 11 each group. (D-E) Differential effects of atgl-1 knockdown by RNAi on peroxisome- and LD-related phenotypes of alh-4(-) worms. (D) Representative images of LDs labeled with DHS-3::mRuby (hj200) in the second intestinal segment. (E) Quantification of the average LD diameter in the second intestinal segment. *p < 0.05, **p < 0.01, ***p < 0.001, ****p < 0.0001, the actual p-value is displayed when p is between 0.05 and 0.1, n.s. (not significant) p > 0.1 (two-way ANOVA with Tukey’s multiple comparisons test). (F) Representative images showing tagRFP::DAF-22 (hj234) in alh-4(-) worms that were subject to control (L4440) or atgl-1 RNAi (left). Quantification of tagRFP::DAF-22 fluorescence (right). n = 8 for each group. (G) As in (F), but with hypodermal peroxisomes labeled with tagRFP::PTS1(hjsi486) (left). Quantification of peroxisome number normalized with lengt [file pgen.1009635.s004.tif]

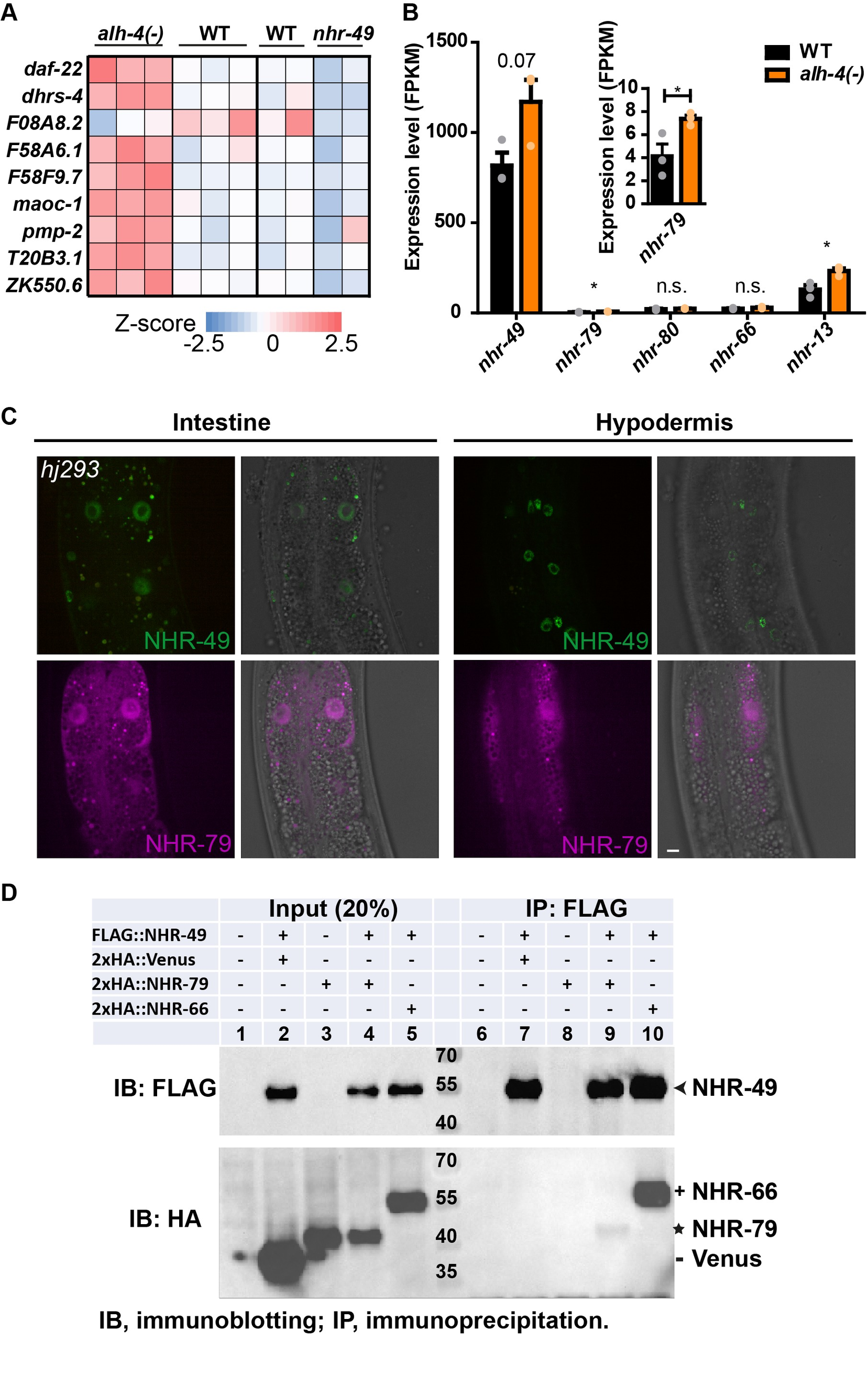

Supplement: S5 Fig — (A) Heatmap showing the up-regulated genes (fold change > 2 and p-value < 0.05, negative binomial test) in alh-4(-) worms in the peroxisome pathway are generally down-regulated in nhr-49(lf) worms. FPKM value is converted to Z-scores. High expression is indicated with red, while low expression is indicated with blue. The data for the WT and nhr-49 groups on the right was extracted from GSE107799 at Gene Expression Omnibus. (B) Histogram showing the mRNA levels of nhr-49, nhr-79, nhr-80, nhr-66 and nhr-13, from RNA sequencing of wild type (WT) and alh-4(-) worms. n = 3 for each group. In the plot, mean ± SEM of each group is shown. *p < 0.05, **p < 0.01, ***p < 0.001, ****p < 0.0001, the actual p-value is displayed when p is between 0.05 and 0.1, n.s. (not significant) p > 0.1 (two-tailed unpaired Student’s t-test). (C) Visualization of NHR-49::GFP (hj293) from the endogenous locus (top) and the visualization of mRuby::NHR-79 (hjEx26[nhr-79p::nhr-79] (bottom). Both NHR-49::GFP and mRuby::NHR-79 could be detected in the nuclei of intestinal and hypodermal cells. Scale bar = 5μm. (D) Co-immunoprecipitation of NHR-49 and NHR-79. The predicted ligand binding domain of NHR-49B(87-476aa), NHR-79A(156-463aa) and NHR-66A(191-577aa) were transiently expressed as FLAG- or HA-tagged proteins in HEK293T cells. HA::Venus was expressed as a negative control. At least two experiments with independent biological samples were performed. A representative blot is shown. 20% input of each sample was loaded. (TIF) [file pgen.1009635.s005.tif]

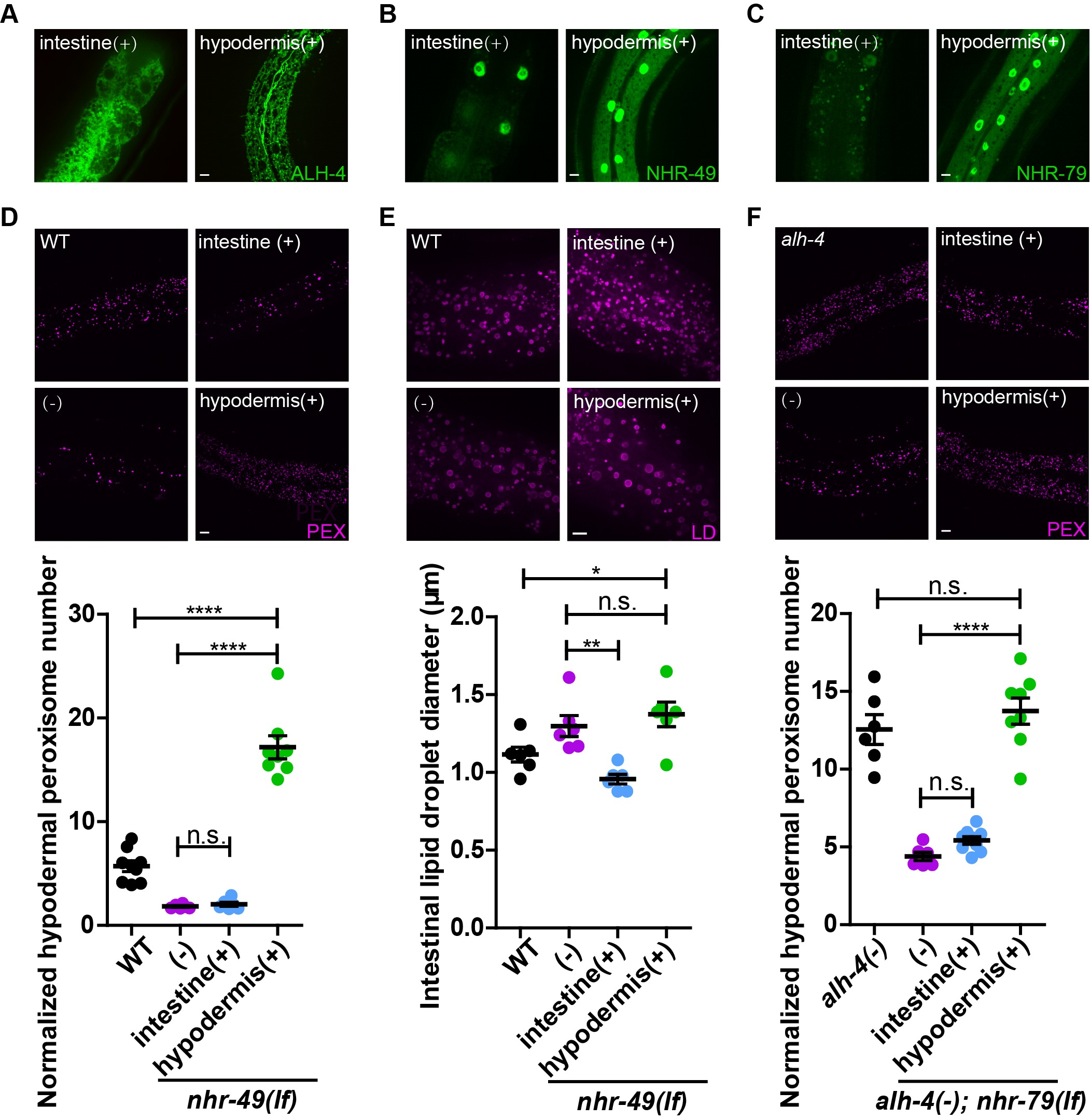

Supplement: S6 Fig — (A) Representative images showing the intestinal specific expression of GFP::ALH-4 in worms carrying hjSi501(left) and the hypodermal specific expression of GFP::ALH-4 in worms hjSi500 (right). (B) Representative images showing the intestinal specific expression of NHR-49A/B::GFP in worms carrying hjSi531 (left) and the hypodermal specific expression of NHR-49A/B::GFP in worms carrying hjSi537 (right). (C) Representative images showing the intestinal specific expression of GFP::NHR-79A in worms carrying hjSi553(left) and the hypodermal specific expression of GFP::NHR-79A in worms carrying hjSi539 (right). (D) Representative images showing hypodermal peroxisomes labeled with tagRFP::PTS1 (hjSi486) (top). Quantification of hypodermal peroxisome number in wild type (WT) and mutant worms of indicated genotypes. n ≥ 6 for each group. (E) As in (D), except with intestinal lipid droplets labeled with DHS-3::mRuby (hj200) (top). Quantification of lipid droplets in the second intestinal segment (bottom). n = 6 for each group. (F) As in (D), except with WT and alh-4(-) and nhr-79(-) mutant worms of indicated genotypes. Quantification of hypodermal peroxisome number (bottom). n ≥ 6 for each group. In each plot, mean ± SEM of each group is shown. For all statistical tests, *p < 0.05, **p < 0.01, ***p < 0.001, ****p < 0.0001, the actual p-value is displayed when p is between 0.05 and 0.1, n.s. (not significant) p > 0.1 (one-way ANOVA with Tukey’s multiple comparisons test). In all plots, mean ± SEM of each group is shown. PEX, peroxisome; LD, lipid droplet. Scale bar = 5μm. (TIF) [file pgen.1009635.s006.tif]
