# Supplementary material for: Nuclear receptors NHR-49 and NHR-79 promote peroxisome proliferation to compensate for aldehyde dehydrogenase deficiency in C. elegans
Source: PLoS Genet. 2021 Jul 8;17(7):e1009635. doi: 10.1371/journal.pgen.1009635 (PMC8291716; doi:10.1371/journal.pgen.1009635)
Supplement: S1 Text — (DOCX) [file pgen.1009635.s011.docx]

**S1 text**

Determinants of organelle specific targeting of ALH-4 isoforms

We determined if each of the three alternative C-termini of ALH-4 was responsible for its targeting to distinct organelles. To this end, we generated single-copy transgenes that placed the expression of GFP::ALH-4A, GFP::ALH-4B or GFP:ALH-3C under the control of the ubiquitous *dpy-30* promoter (S2A Fig). As a result, we were able to observe the localization of each isoform in the intestine and hypodermis at the same time. We found ALH-4A at mitochondria and peroxisomes in both tissues (S2B-E Fig). In contrast, ALH-4B was found primarily at the ER in the intestine (S2F Fig). However, we could not observe the association of ALH-4B with specific organelles in the hypodermis (S2G Fig). Finally, ALH-4C showed tissue specific targeting. In the intestine, ALH-4C was found at mitochondria and LDs (S2H-I Fig). However, it was only found at mitochondria in the hypodermis (S2J Fig). As a complementary approach, we generated three additional single-copy transgenes that drove the ubiquitous expression of GFP fused to the ALH-4 transmembrane helix and one of the three alternative C-termini (S2L Fig). In both intestine and hypodermis, we found that the C-terminus of ALH-4B was sufficient to target GFP to the ER (S2M-N Fig). In contrast to the full length protein, the C-terminus of ALH-4A could only target GFP to mitochondria, while the C-terminus of ALH-4C to mitochondria and the ER. Therefore, the targeting of full-length ALH-4A to peroxisomes, and ALH-4C to LDs appeared to require additional features beyond their C-termini. These features may be important for steering the respective isoforms directly to the second location. Alternatively, they may be required for the transfer of mitochondrial ALH-4 to peroxisomes or LDs via yet to be defined contact sites. Taken together, we conclude that the C-terminus of each ALH-4 isoforms serves as a tail anchor to membranes of distinct organelles.
