# Supplementary material for: Nuclear receptors NHR-49 and NHR-79 promote peroxisome proliferation to compensate for aldehyde dehydrogenase deficiency in C. elegans
Source: PLoS Genet. 2021 Jul 8;17(7):e1009635. doi: 10.1371/journal.pgen.1009635 (PMC8291716; doi:10.1371/journal.pgen.1009635)
Supplement: Attachment — Submitted filename: Response_PLoSGen_7May.docx [file pgen.1009635.s012.docx]

Reviewer #1: In this manuscript, the authors report that nuclear receptors NHR-49 and NHR-79 play a role in regulating peroxisome proliferation to counteract aldehyde dehydrogenase deficiency. They first demonstrated in C. elegans that loss of aldehyde dehydrogenase alh-4 supresses lipid droplet (LD) expansion in daf-22 mutant worms. Next, they showed that ALH-4 associates with different organelles including peroxisomes through membrane anchoring. They also found a significant upregulation of peroxisome-related genes in alh-4(-) worms and that peroxisome proliferation was independent of ATGL-1 directed lipolysis. Next, they identified transcription factors NHR-49 and NHR-79 to be required in response to the lack of ALH-4 and that both regulate peroxisome proliferation. Their data suggest that intestinal ALH-4 regulates hypodermal peroxisome proliferation while NHR-49 and NHR-79 act cell-autonomously.

Overall, the experiments were well designed, executed, and included detailed analysis. The conclusions of the manuscript offer important perspectives on the role of ALH-4, NHR-49, and NHR-79 in peroxisome proliferation. However, there are few points that should be addressed to strengthen the authors’ manuscripts. I recommend the authors to perform additional experiments, to consider alternative explanations to rationalize their data, and/or to more/remove some of their experiments. I highly recommend the publication of the manuscript in PLOS genetics once my points are addressed by the authors.

Major points to address:

(1) The forward genetic screen for suppressor mutants that reversed the daf-22 mutant phenotypes seems to be a high labor-intensive screen if phenotype was monitored by SRS and that the difference between WT and daf-22 is not that obvious. No details are provided in the method section. How many clones were screened?

*The forward genetic screen for mutants that suppressed lipid droplet expansion of daf-22 mutant worms was done by staining mutagenized worms with C1-BODIPY-C12. Mutant worms were examined using a dissecting fluorescence microscope instead of SRS. F2 worms from about 16,000 F1 worms were screened. Accordingly, the genetic screen was unlikely to be saturated.*

The change in the manuscript:

In the method part for ***C. elegans* genetic screen**

From

“The *daf-22(ok693)* mutant worms were mutagenized with Ethyl Methane Sulfonate (EMS) for suppressors of the expanded LD phenotype.”

to

“The *daf-22(ok693)* mutant worms were mutagenized with Ethyl Methane Sulfonate (EMS) for suppressors of the expanded LD phenotype, as indicated by C1-BODIPY-C12 staining.”

(2) The data of LDs in alh-4(hj221) is not reported. The authors only show alh-4 RNAi in Fig. S1B. LDs in alh-4(hj221) must be reported as it is used in subsequent experiments where it is assumed that the mutant suppress daf-22 LDs phenotype.

*The data about LDs in alh-4(hj221) (also labeled as alh-4(-) in short) is displayed in the figures: 5F, 6A, S2O and S2Q, where both WT and alh-4(-) are used as the control groups. In all these replicates, the LDs of alh-4(-) are significantly smaller than that of WT. To avoid data redundancy, we did not provide additional figures to show the LD phenotype of alh-4(hj221) specifically.*

*Similar to alh-4(hj29) or alh-4 RNAi, alh-4(hj221) also suppresses LD expansion in daf-22 mutant worms as shown in Figure I:*

Figure I. *alh-4(hj221)* suppresses lipid droplet expansion of *daf-22(ok693)* mutant worms. (A) Representative images of the second intestinal segment of L4 worms. Lipid droplets (LDs) are labeled with mRuby::DGAT-2*(hjSi112)*. Enlarged LDs are marked by arrowheads. Scale bar = 5μm. (B) Quantification of lipid droplet diameter in the second intestinal segment. n = 7 for both groups. Two-tailed unpaired Student’s t-test was applied. ****p < 0.0001 and mean ± SEM of each group is shown.

The change in the manuscript:

The p-value label between WT and *alh-4(-)* has been added to Fig. 6A.

From	To:

(3) The authors reported triglyceride (TAG) levels in WT and alh-4(-) worms. I would have expected the authors to include daf-22 and daf-22; alh-4(-) mutants to their analysis.

*We have reported the TAG levels of daf-22 mutant worms previously, using gas chromatography–mass spectroscopy (Journal of Cell Biology, vol. 198, no. 5, 2012, pp. 895–911, doi:10.1083/jcb.201201139). Accordingly, daf-22 mutant worms have a 50% increase in TAG levels comparing to WT. More importantly, we showed that the difference in TAG levels correlated well with LD size measurement of fixed samples (Oil-Red-O) and live animals (Red-BODIPY-C12 and mRuby::DGAT-2).*

*In this paper, beyond TAG measurement, we focused our liquid chromatography-mass spectrometry (LC-MS) analysis on a broader range of lipid species in WT and alh-4(-) worms, including fatty aldehydes. The reduction of LD size in alh-4 mutant worms, measured by SRS and mRuby::DGAT-2, correlated well with the reduction of TAG measured by LC-MS. Thus, we were confident in the quantitative difference in other lipid species as detected by LC-MS. Based on the similarity of LD size in both alh-4(hj29) and daf-22(-); alh-4(hj29) worms (Fig. 1D), our LC-MS analysis implied that there should be a corresponding decrease in TAG content in daf-22(-); alh-4(hj29) worms as well.*

*We agree that the analysis of daf-22(-); alh-4(hj29) and daf-22(-) by LC-MS will enhance the comprehensiveness of our study. However, we have difficulty to get the experiment done within one month as the LC-MS instrument in our core facility is only available for lipid analysis once a month.*

(4) Pumping rate are abnormally high in Fig. S1F. It should be around 200-250 pumps/min in WT fed normal diet. Why are the rates double than most publications?

*We also noticed that the pumping rate of N2 WT worms reported by us is higher than that reported by others previously. But it should not be due to error in counting as pharyngeal pumping of individual worms was video recorded and played at 0.3x speed for counting.*

Also, the authors should include more than 7 worms per condition.

*We repeated the experiment and included 10 worms for each group. The average pumping rate for WT is 326 pumps/min, still about 30% higher than that of the pumping rate reported (Fig. II).*

*Figure II.* Quantification of the pharyngeal pumping rate of WT, *alh-4(hj29)* and *alh-4(hj221)* worms. n = 10 for each group. Two-tailed unpaired Student’s t-test was applied.

*Though the pumping rate we reported seemed higher than expected, there was no significant difference in the pharyngeal pumping rate between alh-4(-) and WT in both replicates, indicating the reduced lipid storage in alh-4(-) is probably not due to the difference in food intake.*

*The high pumping rate we recorded was probably due to the temperature at which the videos were recorded. As we do not have a temperature-controlled platform for our microscope, the recording of the pharynx pumping is normally done at room temperature, which varies. The initial dataset was collected in the middle of the summer when the room temperature was higher than 25^o^C. The new dataset was acquired recently when the room temperature was 25^o^C. It is possible that the pharyngeal pumping rate increases with temperature.*

*Regardless of temperature change, our experiments for WT and mutants were done in parallel to make sure that all worms were subject to the same environmental factors. The worms were kept at 20℃ in the same box. They were taken to the bench 2 hours before video recording to be acclimatized to the room temperature. During the video recording: we recorded the videos for two WT worms on the same plate, then two alh-4(-) worms and finally two alh-4(hj29) for each cycle and repeat the cycle with new worms. The counting was done when videos were played back at 0.3x speed to ensure accuracy. In addition, the videos were not labeled with strain names to avoid bias.*

The change in the manuscript:

 to

(The panels in Figure S1 have been rearranged according to the suggestion of reviewer #1. So, the data in Fig. S1F is not in Fig. S1E.)

In the corresponding figure legend . : “n = 7 for each group.” is replaced by “n = 10 for each group.”

(5) I find the section where the authors justify why GFP knockin strain retains normal ALH-4 function very confusing (lines 163-169). The authors argue using data that is described later in the text. I do not see the point to argue that their ALH-4 fusion protein is functional at this point by referring to data that is shown later in the text. This section would be more appropriate elsewhere either after Fig. 3 or in the discussion.

*The change in the manuscript:*

“This GFP knockin strain, *alh-4(hj179)*, retained normal ALH-4 function for two reasons. First, the knockin strain showed no significant difference in the intestinal LD size when compared with wild type worms (Fig 3E-F). Second, LD expansion was unperturbed when daf-22 was mutated, unlike what we observed in alh-4(-) worms.”

*To:*

*“*This GFP knock-in strain, *alh-4(hj179)*, retained normal ALH-4 function because LD expansion was unperturbed when daf-22 was mutated, unlike what we observed in daf-22; alh-4(-) worms.*”*

(6) The authors went into the distance to determine the subcellular localization of GFP::ALH-4 by using different organelle markers. I think this is very important and informative. However, I would like to see higher magnification to appreciate the localization of ALH-4 within cells.

*Thank you for appreciating our study of the subcellular localization of GFP::ALH-4. And we are sorry that the images shown in the manuscript are already the best resolution we can achieve with an existing spinning-disk confocal microscope.*

*We found that GFP::ALH-4 is associated with many organelles (Fig. 2). In rescue experiments with single-copy transgenes, we found that each ALH-4 isoform, which associates with distinct organelles can efficiently rescue the alh-4 mutant phenotypes: smaller LDs and more hypodermal peroxisomes than wild type worms (Fig. S2O-R). Therefore, the targeting of ALH-4 to a specific organelle is not essential for its function in regulating organismal lipid metabolism. As a result, we focused our effort in other aspects of the project instead of attempting super-resolution microscopy.*

*Besides the fluorescence images, the strong correlation of fluorescence intensity profiles between GFP::ALH-4 and different organelles markers (Fig. 2) ascertained the localization of GFP::ALH-4. Specifically, we have used the markers to label the membrane of lipid droplets, mitochondria, the ER and peroxisomes. We were slightly surprised by the localization of ALH-4C isoform to LDs (FigS2H & K). Notably, ER proteins that are enriched in the ER-LD interface may appear to surround LDs (Journal of Cell Biology, vol. 198, no. 5, 2012, pp. 895–911, doi:10.1083/jcb.201201139). Nevertheless, ALH-4 is one of the 100 most abundant lipid droplet proteins found in a proteomic analysis of C. elegans lipid droplets (Vrablik, Tracy L., et al. “Lipidomic and Proteomic Analysis of Caenorhabditis Elegans Lipid Droplets and Identification of ACS-4 as a Lipid Droplet-Associated Protein.” Biochimica et Biophysica Acta - Molecular and Cell Biology of Lipids, vol. 1851, no. 10, Elsevier B.V., 2015, pp. 1337–45, doi:10.1016/j.bbalip.2015.06.004).*

(7) The number of worms should be clearly reported for all quantification based on fluorescence microscopy images. For instance in Fig. S4A,B, how many worms are reported per condition. The authors should distinguish technical from biological replicates in the figure as they are both reported with the same colour.

*Thank you for the reminder. We have modified the figure so that data points from different biological replicates are clearly indicated and the number of worms measured in each experiment reported in the figure legend of Fig. S4.*

The change in the manuscript:

To:

The sentence “Three independent biological samples of each genotype were tested. Each dot represents one of three technical replicates of each biological sample.” has been modified.

Add “The total number of worms measured in 9 biological replicates were 4, 13, 7 for WT and 34, 8,12 for *alh-4(-)* in the first run; 8, 10, 36 for WT, 19, 12, 13 for *alh-4(-)* in the second run and 14, 21, 19 for WT and 25, 10, 11 for *alh-4(-)* in the third run. Data points are color-coded to indicate results from specific experiments.” in the figure legend for Fig. S4A.

Add “The total number of worms measured in 9 biological replicates were 41, 27, 35 for WT and 32, 28, 33 for *alh-4(-)* in the first run; 16, 21, 22 for WT and 17, 24, 35 for *alh-4(-)* in the second run and 25, 28, 27 for WT and 29, 28, 29 for *alh-4(-)* in the third run.” in the figure legend for Fig. S4B.

(8) It is not clear to me which genes are upregulated or if all of them are upregulated in Fig. 4B.

*Fig. 4B shows the peroxisomal fatty acid oxidation pathway. Not all genes encoding proteins involved in this process were upregulated in alh-4(-): some of them showed no difference between WT or alh-4(-) at the mRNA level, the majority showed upregulation but none showed significant reduction in alh-4(-). As for those genes whose expression showed a difference between WT or alh-4(-), we displayed their expression level in Fig. 4C. They were all upregulated.*

*We summarized the expression level of those genes reported to play a role in peroxisomal fatty acid β-oxidation in Fig. III (the raw data can be found in the worksheet named ‘peroxisomal_oxidation’ in the supplementary dataset 2). The information overlaps with that in Fig. 4C. We hope such a figure can provide a better view of this process.*

Figure III. Left: Schematic representation of the peroxisomal fatty acid β-oxidation pathway as in Fig. 4B. Right: Heatmap showing the expression level of genes encoding enzymes for peroxisomal fatty acid β-oxidation in WT and *alh-4(-)* worms. Two-tailed unpaired Student’s t-test was applied., *p < 0.1, **p < 0.01, ***p < 0.001. Not all genes in this process are differentially expressed but those showing significant difference in their expression levels are consistently upregulated in *alh-4(-)*, comparing to WT.

No corresponding change in the manuscript.

(9) The interaction seems rather very weak between NHR-49 and NHR-79 and the band corresponding to NHR-79 in the IP seems much higher in MW compared to input.

*We agree that the interaction between NHR-49 and NHR-79 under our experimental condition is weak. But such a result is consistent with that reported in the yeast two hybrid experiment: the interaction strength between NHR-49 ligand binding domain (LBD) and NHR-79 was estimated to be only 12, which was much weaker than that between NHR-49 and NHR-66 at 457 (the data can be found in Table S2b in the paper: Pathare, Pranali P., et al. “Coordinate Regulation of Lipid Metabolism by Novel Nuclear Receptor Partnerships.” PLoS Genetics, vol. 8, no. 4, 2012, doi:10.1371/journal.pgen.1002645.). Therefore, our co-IP results provide an independent validation of the relatively weak interaction between NHR-49 and NHR-79.*

*Such a weak interaction may be due to a lack of cognate ligand since the dimerization of many nuclear receptors are known to be regulated by ligand binding. The dimerization of nuclear receptors is mediated by ligand binding domain (LBD). Ligand binding induces the conformational change LBD and regulates the strength and dynamics of such interaction (Tamrazi, Anobel, et al. “Estrogen Receptor Dimerization: Ligand Binding Regulates Dimer Affinity and Dimer Dissociation Rate.” Molecular Endocrinology, vol. 16, no. 12, 2002, pp. 2706–19, doi:10.1210/me.2002-0250). The identification of NHR-49 or NHR-79 ligands is beyond the scope of our current study.*

*We did not notice an obvious difference in the MW of the NHR-79 band in the input and the elution (Fig. S5D, comparing lane 4 and lane 9). Furthermore, in this set of experiment, each protein has a distinct MW and therefore the NHR-79 band identified in the eluted sample could not result from contamination by other proteins. The result for a second co-IP experiment is shown below (Fig. IV). Additional samples were tested. We included another truncated form of NHR-79 to confirm the interaction between NHR-79 and NHR-49. In our manuscript, we chose to only show the result for the shortest form of NHR-79 to avoid redundancy.)*

Figure IV. Co-immunoprecipitation of NHR-49 and NHR-79. The predicted ligand binding domain of NHR-49B (87- 476aa), NHR-79A(156-463aa), NHR-79A(125-463aa) and NHR-66A(191- 577aa) were transiently expressed as FLAG- or HA-tagged proteins in HEK293T cells. HA::Venus was expressed as a negative control.

Have the authors tried the reciprocal IP by IPing HA?

*Yes. We have tried reciprocal IP twice by enriching HA::NHR-79 with anti-HA beads. Our current experimental conditions precluded us from detecting FLAG::NHR-49 so far. It could be due to insufficient enrichment of HA::NHR-79A(156-463aa) with anti-HA beads or the binding of HA:: NHR-79A(156-463aa) with beads results in steric effects that block the interaction between NHR-79 with NHR-49. However, we emphasize that our co-IP results are consistent with that obtained by independent yeast 2-hybrid experiments (Pathare, Pranali P., et al. “Coordinate Regulation of Lipid Metabolism by Novel Nuclear Receptor Partnerships.” PLoS Genetics, vol. 8, no. 4, 2012, doi:10.1371/journal.pgen.1002645).*

(10) Although reporting NHR-49 is required for reproduction of ALH-4 deficient worms is interesting. I do not see how it fits with previous results and how it adds to the understanding of the mechanism. In my view, it should be moved to supplementary data and certainly removed as the last sub-section of the result section.

*We agree with the reviewer partially.*

*The strong defects observed in alh-4(-); nhr-49(lf) show that the activity of NHR-49 becomes essential in alh-4(-). This result suggests the activation of NHR-49 and the subsequent induction of genes encoding peroxisomal enzymes in alh-4(-) is a protective response against the defect of fatty aldehyde metabolism. In addition, the severe synthetic phenotype of alh-4(-); nhr-49(lf) is a piece of strong evidence to show the genetic interaction between alh-4 and nhr-49. Further investigation is needed to pinpoint the molecular mechanism, which is beyond the scope of our current study.*

*In addition to being sterile, alh-4(-); nhr-49(lf) worms in fact grow much slower comparing to WT, alh-4(-) or nhr-49(lf) (Fig. 8B in the updated manuscript). In addition, the double mutant worms also have a much thinner and more transparent body. Thus, loss of nhr-49 affects the development of alh-4(-) worms in addition to their reproduction.*

*To better emphasize the pleiotropic phenotypes of alh-4(-); nhr-49(lf) worms, we prefer keeping Fig. 8A while adding Fig. 8B to show the growth delay phenotype.*

The change in the manuscript:

Fig. 8B has been added and Fig. 8B renamed Fig. 8C. And the figure legend is also updated correspondingly.

From

to

The title of Figure 8 is renamed: “NHR-49 is required for the growth and reproduction of *alh-4(-)* worms.”

Figure legend for new Fig. 8B has been added:

“(B) Quantification of body length of wild type (WT), mutant worms of indicated genotypes 44 hours after being hatched out (left). The representative bright field images of worm body of indicated genotypes (right). Scale bar = 100μm. n = 26 for WT, *alh-4(-)* and *nhr-49(lf)*. n = 29 for *alh-4(-); nhr-49(lf)* and *alh-4(-); nhr-49(lf);Ex[alh-4(+)]*.*”*

The description of Fig. 8B has been added to the text as follows.

“Furthermore, *alh-4(-)*; *nhr-49(lf)* double mutant worms could be distinguished from *alh-4(-); nhr-49(lf);Ex[alh-4(+)]* and single mutant worms as they show obvious growth delay (Fig 8B). In contrast, the reproduction of *alh-4(-)* worms was not strictly dependent on NHR-79. Accordingly, *alh-4(-)*; *nhr-79(lf)* mutant worms remained fertile, although their brood size was reduced in comparison to that of *alh-4(-)* or *nhr-79(lf)* single mutant worms (Fig 8A). We conclude that NHR-49 is a key factor that supports the development and reproduction of *alh-4* deficient worms, in part because of its broad role in regulating peroxisome proliferation and fat storage.”

“Fig. 8B” in the text has is replaced with “Fig. 8C”.

Minor points to address

(1) I suggest the authors to change the title of line 113 to something more meaningful.

*Thanks for the suggestion and we have changed the title in the revised version.*

The change in the manuscript:

The subtitle title “Molecular cloning of *alh-4*” has been replaced with “Loss of *alh-4* function suppresses lipid droplet expansion.”

(2) Define LDs in line 115.

*Thanks for the reminder.*

The change in the manuscript:

“LDs” has been replaced with “lipid droplets (LDs)”

(3) Do the authors meant “sequencing” instead of “molecular cloning” in line 121?

*Yes.*

The change in the manuscript:

“Molecular cloning” has been replaced with “Following genetic mapping, targeted sequencing of the alh-4”.

(4) Fig. S1C should not appear after Fig. S1D-E. This is a recurrent issue through the manuscript and should be fixed. Figures and figure panels should appear in chronological order through the manuscript.

*Thanks for the suggestion and the sequence of the figure S1 has been rearranged to be in chronological order. The figure legend has been rearranged correspondingly.*

The change in the manuscript:

S1C has been named S1F;

S1D-E has been named S1C-D;

S1F has been named S1E.

Another example is Fig. S3A that is cited before Fig. S2.

*Thank you for the suggestion but we prefer to keep it unchanged for it is a tradeoff between the display of data in chronological order or in a neat layout. We choose the layout in this case for the following reasons.*

*S3A shows the phylogram tree of fatty aldehyde dehydrogenase. We would like to introduce ALH-4 briefly by showing its family tree before showing the subcellular localization. But Fig. 2 and Fig. S2 already occupies one page and more than two pages, respectively. Therefore, we did not want to add more panels to them. Instead, we added the phylogram tree to Fig. S3 as it is more related to other content in the same figure, which is about the structure-function study of ALH-4 based on its sequence similarity with its human ortholog ALDH3A2.*

No corresponding change in the manuscript.

Fig. 3D, F are cited before Fig. 2B-F.

*We failed to find the problem in the manuscript.*

*Fig. 2B-F was first cited at line 164, while 3D was at line 195; 3F at line 199. It seems they are already in the right order in the manuscript.*

No corresponding change in the manuscript.

(5) The sentence “In agreement with our…” in lines 138-140 is repetitive as it is already reported above.

*We agree that the sentence is a repetition. We deleted that sentence in the revised version.*

The change in the manuscript:

The sentence “In agreement with our observations with SRS, ALH-4 deficiency reduced the average intestinal LD size of wild type and *daf-22* mutant worms (Fig 1C-D).” has been deleted.

(6) The authors refer to alh-4 isoforms in lines 157-158 but Fig. 2A does not include any isoforms.

*In Fig. 2A, we showed how alternative splicing could generate different isoforms instead of the gene structure of each isoform. Please note that those isoforms only differ at the 3’ end. We think such a display can save space while keeping the information of the gene structure of alh-4 isoforms. We have modified the figure to make the alternative splicing pattern more obvious in Fig. 2A.*

*In addition, the isoform-specific sequences are labeled in Fig. S1A.*

The change in the manuscript:

The figure 2A:

Is changed to:

Reviewer #2: This manuscript by Dr. Ho Yi Mak and colleagues identifies a cross-tissue genetic pathway responsible for maintaining body fat mass in C. elegans. The pathway, involving intestinal ALH-4 and hypodermal peroxisomal proliferation downstream of NHR-49 and NHR-79 suggests that the latter responds to and is critical to the defense against excess levels of fatty aldehydes. It is particularly curious that the pathway would not be fully cell autonomous, that is excess aldehydes not dealt with in the tissues that they are generated in, and this adds to the interest of the work. The authors utilize state-of-the-art genetic and genomic methodologies that add strength to the conclusions put forward. In general, I find the conclusions sound, interesting, and robustly deduced. I do have modest concern about the interest level of the topic the broad readership of PLoS Genetics. Additionally, in some areas, the manuscript suffers from methodological and statistical inconsistencies that could be rectified to further strengthen conclusions:

Comment 1: It looks as though for fat mass, the relationship of alh-4 and daf-22 are parallel rather than epistatic; for lipid droplet alternatively the suppression looks epistatic. Please discuss in the text (Fig 1B&D).’

*We agree with you. Our results could be viewed in genetic or molecular terms.*

*In molecular terms, ALH-4 and DAF-22 work in parallel: ALH-4 affects the transcription of genes in peroxisomal fatty acid oxidation, in parallel of DAF-22. In genetic terms, alh-4 acts downstream or in parallel of daf-22, as loss of alh-4 function fully suppresses the lipid droplet expansion in daf-22 mutant worms and makes lipid droplets as small as that in alh-4 mutant worms (Fig. 1C-D). We noticed a slight increase of lipid droplet number, though not significant, in daf-22; alh-4(hj29) comparing to alh-4(hj29) (Fig.1E), indicating that in alh-4 mutant worms, loss of function of function of daf-22 may increase fat mass by increasing the number of lipid droplets.*

*The regulation of lipid droplet size in alh-4 mutant worms relies on the transcriptional response independent of daf-22. In our study, the downstream transcriptional response mediated by NHR-49 or NHR-79 can induce peroxisomal fatty oxidation and result in less lipid storage in alh-4(-). Reduction of lipid storage can be shown as reduced lipid droplet size, decreased lipid droplet number or both. In alh-4 mutant worms, the reduction of lipid storage is shown as the shrinkage of lipid droplets while no defects in the lipid droplet number (Fig. 1C-E).*

The change in the manuscript:

Modification in the Discussion:

“We originally cloned *alh-4* based on the observation that *alh-4* loss of function alleles suppressed fat accumulation and LD expansion in *daf-22* mutant worms, suggesting that *alh-4* acts downstream or in parallel of *daf-22*. Our subsequent molecular analysis clearly indicates the latter…… Our results are consistent with the notion that *alh-4* deficiency specifically activates peroxisomal fatty acid β-oxidation pathways that act in parallel of the DAF-22 pathway. *”*

Comment 2: Figure legend 1 says that two-way ANOVA with Tukey’s post hoc test was used, but then the figure legend also says t-tests were applied. Please clarify. Which is it? It is very important that multiple hypotheses be corrected for in the statistical analyses, particularly when multiple measurements are conducted as in 1B, D, E, and F.

*In the figure legend of Fig. 1, it is stated that “Two-way ANOVA with Tukey’s multiple comparisons test was applied in B, D & E.” after (E) and “Two-tailed unpaired Student’s t-test was applied.” after (F).*

*We applied different statistical tests according to the number of factors involved in each set of experiments and we will further clarify it in the figure legend.*

*We do agree with that ‘multiple hypotheses be corrected for in the statistical analyses’. Applying a suitable statistical test based on the experimental design is also important for the appropriate data interpretation*. *Thus, in 1B, 1D, 1E, where two factors (alh-4 and daf-22) were involved in the study, two-way ANOVA was applied for statistical analysis and Tukey’s post hoc test was applied to correct the p-value for multiple comparisons. While in Fig. 1F, each statistical analysis only focusses one lipid species. Thus, two-tailed unpaired Student’s t-test was applied. Though the correction of the p-value can be applied, considering that the total number of the t-test is only 11, such a correction may not be necessary, and therefore we prefer to display the p-value from Student’s t-test directly.*

The change in the manuscript:

In figure legend for Fig. 1, the sentence “Two-tailed unpaired Student’s t-test was applied.” is replaced by “Two-tailed unpaired Student’s t-test was applied **in F***”,* to emphasize Student’s t-test was only applied in F while not in other figures in Fig. 1.

Comment 3: what does the X axis represent in figs 2B-F (right panels)? distance? Percentage of total distance? Microns? Please clarify.

*Thank you for the reminder.*

*The X axis in the previous version is the distance in the unit of pixel. In the latest version we have converted the unit of the distance to μm in Fig. 2B-F.*

*In addition, in Fig. 6D, F & H, the label for the x axis “Z position (focal plane)” has been added.*

The change in the manuscript:

In Fig.2:	to

In the Fig. 6 D,F&H:

“Z position (focal plane)” for X-axis label has been added.

Also, is it possible to show higher magnification images for co-localization?

*We are sorry that the images we show are already the ones with the best resolution obtained with our confocal microscope. For better resolution, we need to turn to electron microscopy or super-resolution microscopy. Unfortunately, both are impractical to perform within one month.*

No corresponding change in the manuscript.

Is ALH-4 predicted to have an n-terminal mito targeting signal that the authors interfered with by knocking in the GFP at the n-terminus?

*Many mitochondrial proteins are transported to mitochondria with an N-terminal mitochondrial targeting signal (Habib, Shukry J., et al. “Analysis and Prediction of Mitochondrial Targeting Signals.” Methods in Cell Biology, vol. 80, no. 06, 2007, pp. 761–81, doi:10.1016/S0091-679X(06)80035-X.).*

*However, ALH-4 (and its human ortholog ALDH3A2) is a tail-anchored protein with a C-terminal transmembrane domain (Fig. S3B-C, Fig. V) (Chio, Un Seng, et al. “Mechanisms of Tail-Anchored Membrane Protein Targeting and Insertion.” Annual Review of Cell and Developmental Biology, vol. 33, 2017, pp. 417–38, doi:10.1146/annurev-cellbio-100616-060839.). Thus, we chose to fuse GFP to the N-terminus of ALH-4 to prevent any disruption to the C-terminal tail anchor.*

Figure V. A single transmembrane domain is predicted to be at the C-terminus in ALH-4 with TMHMM Server v.2.0(Transmembrane Protein Topology with a Hidden Markov Model: Application to Complete Genomes’, 2001 <https://doi.org/10.1006/jmbi.2000.4315>). The full amino acid sequence of ALH-4A isoform, which shares most the sequence with other isoforms except the last 7 amino acids, is used as the input.

*Furthermore, the C-terminus of either ALH-4A or ALH-4C is sufficient to guide GFP to mitochondria (Fig. S2M&N) while that of ALH-4B can guide GFP to the ER (Fig. S2M&N). These results demonstrate that the membrane anchoring of ALH-4 is primarily dependent on its C-terminus.*

*In addition, the fact that GFP::ALH-4 is localized on mitochondria in the hypodermis (Fig. 2B) also shows that the mitochondrial targeting property of ALH-4 is not affected by the GFP tag at its N-terminus.*

What compartments didn’t GFP::ALH-4 associate with?

*Besides the ER, lipid droplets and mitochondria in the intestine and peroxisomes and mitochondria in the hypodermis, we did not detect ALH-4 in other membrane-bound organelles. For example, ALH-4 did not associate with lysosome related organelles, lysosomes and the Golgi apparatus.*

*Also, GFP::ALH-4 shows tissue specific organelles targeting property. Within one tissue, it can associate with no more than three organelles. For example, GFP::ALH-4 is not found on peroxisomes in the intestine.*

No corresponding change in the manuscript.

The authors find evidence for all compartments having ALH-4 enrichment which could be interesting or, alternatively, confounded.

*We were surprised by the discovery that ALH-4 could target to different organelles. Subsequently, we found that each ALH-4 isoform could be targeted to specific organelles, as a result of isoform-specific C-terminus (Fig. S2A-K). In our knockin strain that expressed GFP::ALH-4 fusion proteins, all isoforms were detected simultaneously. Therefore, our results gave the impression that ALH-4 could associate with many, but not all, organelles.*

*Our initial discovery that different ALH-4 isoforms could localize on different organelles led us to investigate if there is any isoform-specific function. However, subsequent experiments when a single ALH-4 isoform was expressed suggested that each isoform was sufficient to rescue alh-4(-) phenotypes (Fig. S2O-R). As for why ALH-4 evolves to have different isoforms residing on different organelles, it could be that ALH-4 may provide some benefit to those organelles by detoxifying fatty aldehydes locally. But such a benefit may not be essential enough to be revealed under normal culture condition.*

Comment 4: The TM domain deletion experiment (Fig. 3D-F) could have “failed” to rescue because of defective folding of the mutant protein rather than ineffective targeting to the proper cellular location (organelle or to membranes). This possibility should be addressed.

*Thank you and we will address the possibility in the manuscript.*

The change in the manuscript:

The title of the result “Membrane anchoring is required for ALH-4 function” is replaced by “The transmembrane domain and the gate-keeper helix are required for ALH-4 function.

The conclusion at the end of the result part, membrane anchoring is required for ALH-4 function has been edited.

From

“Taken together, our results indicate that membrane anchoring by the transmembrane helix, and the gate-keeper helix are both required for ALH-4 function in vivo.”

To:

“Our results show that the C-terminus containing the transmembrane domain and the gate-keeper helix are both required for ALH-4 function in vivo. It should be noted that our deletion mutants were designed based on the ALDH3A2 structure, in order to prevent the disruption of globular domains. However, we cannot rule out folding defects of these mutants, which might contribute to their functional deficit. Additional structural studies will be required to address this issue further.”

Does the C- terminus harbor glycosylation sites that could be missing when deleted? These also could be involved in proper cellular targeting. The same could be true for the deltaGK mutant.

*We conducted glycosylation site prediction of ALH-4 with NetOGlyc 4.0 (Steentoft, Catharina, et al. “Precision Mapping of the Human O-GalNAc Glycoproteome through SimpleCell Technology.” EMBO Journal, vol. 32, no. 10, 2013, pp. 1478–88, doi:10.1038/emboj.2013.79.). There is no potential glycosylation site at the C terminus of ALH-4 and only one potential site at the N-terminus: Q16. Thus, the deletion of its C-terminus probably would not directly interfere with glycosylation of ALH-4. Nor would the deletion of the gatekeeper region as no potential glycosylation site is predicted within the region.*

*Though, we designed the deletion mutations of alh-4 based on the structure of its human ortholog ALDH3A2, we cannot completely rule out the possibility that the modifications of the protein sequence can yield unexpected changes in protein function. We added the following statement (line 215)*

“It should be noted that our deletion mutants were designed based on the ALDH3A2 structure, in order to prevent the disruption of globular domains. However, we cannot rule out folding defects of these mutants, which might contribute to their functional deficit. Additional structural studies will be required to address this issue further.”

Comment 5: The relationship of ATGL to ALH-4 looks parallel rather than epistatic with regard to lipid droplet diameter in figure S4D-E), although the comparison between WT and alh-4 mutants on atgl-1 RNAi is not shown. Or if it is not significant, then the conclusion may be justified. As such the comparison is important to justify the conclusions.

*Thank you for your reminder. We have added the statistics result in the figure.*

*ATGL-1 and ALH-4 work in parallel in the regulation of the lipid storage*. *We observed an increase in average intestinal LD size, to a similar extent, when atgl-1 was knocked down by RNAi in wild type and alh-4(-) worms (Fig S4D-F). This is confirmed by* *two-way ANOVA analysis (the p-value for the interaction is 0.26, not significant).*

*We attempted to address if fatty acids released by ATGL-1 mediated lipolysis could work as signaling molecules to regulate the transcriptional response in alh-4(-) worms. NHR-49 is a functional ortholog of human PPARα (Peroxisome Proliferator-Activated Receptor (PPAR)-alpha) and PPARα has been reported to be activated by lipolytic products to drive the expression of the genes for fatty acid oxidation (Mottillo, Emilio P., et al. “Lipolytic Products Activate Peroxisome Proliferator-Activated Receptor (PPAR) α and δ in Brown Adipocytes to Match Fatty Acid Oxidation with Supply.” Journal of Biological Chemistry, vol. 287, no. 30, 2012, pp. 25038–48, doi:10.1074/jbc.M112.374041.). Nevertheless, atgl-1 RNAi did not alter daf-22 expression or the abundance of hypodermal peroxisomes (Fig S4F-G). Our results indicate that lipolysis is not required for the NHR-49 dependent transcriptional response toward ALH-4 deficiency.*

*We have rewritten the paragraph describing the result of atgl-1 RNAi to present our results more precisely. Our conclusion that “… lipolysis is not required for the transcriptional response toward ALH-4 deficiency” is still valid.*

The change in the manuscript:

Figure S4E has been modified.

To:

The following paragraph has been rewritten：

“From our gene expression analysis, we noted that *atgl-1*, which encodes the *C. elegans* adipose triglyceride lipase (ATGL) ortholog, was induced in *alh-4(-)* worms (Fig 4B-C). ATGL hydrolyzes a fatty acyl chain from TAG to yield DAG [48]. Fatty acids can then be activated by acyl-CoA synthetases (ACS) to form acyl-CoA before being channeled to anabolic or catabolic pathways, such as β-oxidation. Therefore, the induction of ATGL reduces cellular fat storage. Accordingly, we observed an increase in average intestinal LD size when *atgl-1* was knocked down by RNAi (Fig S4D-F). Furthermore, RNAi against *atgl-1* in *alh-4(-)* worms restored the average LD size to that observed in wild type worms (Fig S4D-E). Our results indicated that the increase in *atgl-1* expression contributed to the reduced fat storage in *alh-4(-)* worms. It is plausible that fatty acids released from LDs by ATGL-1 can serve as signaling molecules that modulate peroxisome proliferation and function in *alh-4(-)* worms. However, we found that RNAi against *atgl-1* did not alter *daf-22* expression or the abundance of hypodermal peroxisomes (Fig S4F-G). Our results indicate that lipolysis is not required for the transcriptional response toward ALH-4 deficiency.”

To:

“From our gene expression analysis, we noted that *atgl-1*, which encodes the *C. elegans* adipose triglyceride lipase (ATGL) ortholog, was slightly induced in *alh-4(-)* worms (Fig 4B-C). ATGL hydrolyzes a fatty acyl chain from TAG to release fatty acids [48]. Fatty acids can then be activated by acyl-CoA synthetases (ACS) to form acyl-CoA before being channeled to anabolic or catabolic pathways, such as β-oxidation. Besides, lipolytic products as well as exogenous fatty acids have been reported to activate PPARs (Peroxisome Proliferator-Activated Receptors) to induce the expression of genes for fatty acid oxidation [49]*.* It is plausible that fatty acids released from LDs by ATGL-1 can serve as signaling molecules that modulate peroxisome proliferation and function in *alh-4(-)* worms. To test this hypothesis, we knocked down *atgl-1* by RNAi in both WT and *alh-4(-)* worms. We observed an increase in average intestinal LD size, to a similar extent, in both cases (Fig S4D-F). Therefore, ATGL-1 appeared to work in parallel of ALH-4. Furthermore, we found that RNAi against *atgl-1* did not alter *daf-22* expression or the abundance of hypodermal peroxisomes (Fig S4F-G). Our results indicate that lipolysis is not required for the transcriptional response toward ALH-4 deficiency.”

Comment 6: Line 311: The loss of NHR-49 but not NHR-79”… should be “partially” corrected. The epistatic relationships with regard to PEX number and DAF-22 expression are much more convincing for epistasis whereas the LD data suggest the relationship is more complex than just nhr-49/79.

We agree with the comment.

The change in the manuscript:

The sentence

“However, the loss of NHR-49 but not NHR-79 corrected the intestinal LD size of *alh-4(-)* worms (Fig 5F-G).”

To

“However, the loss of NHR-49 but not NHR-79 partially corrected the intestinal LD size of *alh-4(-)* worms (Fig 5F-G).”

Comment 7: I am confused on the presentation of results in Figure 5. The authors indicate in the abstract that alh-4;nhr-49 double mutants are inviable, but proceed to analyze them in Figure 5B-G. The legend does not clearly indicate RNAi and the text suggests double mutants are analyzed. This must be clarified.

*Thank you for your reminder. To be more accurate, alh-4; nhr-49 double mutants are* *genetically inviable or sterile. We have replaced the word “inviable” with “sterile”.*

*In Fig. 5, we keep the double mutant worms with an extra-chromosome array expressing GFP tagged ALH-4, alh-4(-); nhr-49(lf); Ex[alh-4(+)] as in the case of Fig. 8A. Based on the fluorescence from GFP::ALH-4, we can distinguish double mutant worms from their siblings that carry Ex[alh-4(+)]. We have modified the figure legend to clarify this point.*

The change in the manuscript:

In the abstract, the sentence “Such a response is required to counteract ALH-4 deficiency since *alh-4; nhr-49* double mutant animals are inviable.” is replaced by “Such a response is required to counteract ALH-4 deficiency since *alh-4; nhr-49* double mutant animals are sterile.”

In the figure legend for Fig. 5, the sentence “An extra-chromosomal array that expressed functional GFP::ALH-4 (*hjEx25*) was used to propagate *alh-4(-)*; *nhr-49(lf)* worms. Based on the fluorescence of GFP::ALH-4, *alh-4(-)*; *nhr-49(lf)* worms with the extra-chromosomal array could be distinguished from those without it. ” has been added.

In the figure legend for Fig. 8, the sentence “Ex[*alh-4(+)*] represents *hjEx25(dpy-30p::GFP:: alh-4).*”is added.

Also unless the double alh-4;nhr-49 is inviable, it is not clear why this animal is not analyzed for the tissue-specific restorations of nhr-49. I do see in figure 8 that the double mutant makes very few progeny. It would have been interesting to see which of the tissue rescues in figure 5E/F also restore progeny production in the double mutant alh-4; nhr-49.

*alh-4(-); nhr-49(lf) are viable but sterile. After studying 50 double mutant worms, we found that only 40% of them could give birth to a few progeny but none of the progeny was fertile.*

*We actually performed tissue-specific rescue of nhr-49. We found that the expression of nhr-49 in either the intestine or the hypodermis can rescue the sterility of the double mutant worms (Fig. VI). Such a result confirms that the sterility phenotype is regulated by nhr-49 and also suggests that the synthetic sterility of double mutant worms results from defects in the metabolism of somatic cells. In addition, we have shown that intestinal ALH-4 activity promotes NHR-49 dependent peroxisome proliferation in the hypodermis (Fig. 6A-D). Taken together, these results suggest a systemic response against stress induced by fatty aldehydes.*

Figure VI. The expression of *nhr-49* in either the intestine or the hypodermis can rescue the sterility of *alh-4(-); nhr-49(lf).* Quantification of brood size of wild type (WT), mutant worms of indicated genotypes. n > 8 for each group. In the plot, mean ± SEM of each group is shown. For statistical tests, ****p < 0.0001, n.s. (not significant) p > 0.1 (one-way ANOVA with Tukey’s multiple comparisons test).

*(This data was collected in parallel of those reported in Fig. 8A. We chose not to present it in Fig. 8A in order to project a simpler message.)*

Minor issues:
I’m not sure if the conclusion is important given the genetic data, but if the upregulation of nhr-49 and nhr-79 at the mRNA level is significant and a point the authors want to substantiate, it should not be done with semiquantitative methodology like extraction of RNAseq data.

*We simply stated our observations from the RNAseq data (with 3 independent biological samples). We were compelled to show results of statistical tests, lest criticisms for omitting them. Nevertheless, we did not intend to over-interpret the difference in mRNA levels in wild type versus alh-4(-) worms. Instead, we would like to draw the readers’ attention to the fact that the mRNA level of nhr-49 was much higher than its partners. Assuming a good correlation between mRNA and protein levels, there could be enough NHR-49 to form heterodimers with different NHRs at the same time.*

*Though in alh-4(-), the mRNA level of nhr-49 and nhr-79 was increased based on the RNAseq data, such an upregulation is very slight. For the expression of nhr-49, the p-value is 0.07. We agree that it is hard to say if such a difference is really significant without further repeats of the experiment. For nhr-79, its expression level is too low (less than 10 FPKM) for us to make a strong conclusion. Therefore, we favor the notion that the upregulation of NHR-49/NHR-79 activity is probably not due to the change in their expression levels. Instead, post-transcriptional regulation, such as ligand binding may play a role to regulate the activity of NHR-49 or NHR-79.*

“number of progenies” should probably be “number of progeny”. In figure 8 and line 378, etc.

The manuscript has been corrected based on your suggestion.

“number of progenies” has been replaced with “number of progeny” in Fig. 8A, lines 378, 381, 573, and Fig. S1C.

Reviewer #3: Mak and colleagues present a manuscript that describes a new genetic relationship between nhr-49 and nhr-79 in response to loss of the aldehyde dehydrogenase alh-4. This is a well designed and executed study that documents high quality data of the peroxisomal response to these genetic mutations. I found no issues with the methodology, appropriate statistics were used in most experiments, and the overall results are compelling. That said, I believe that there are three major concerns that should be addressed prior to acceptance (two of which can be done with simply editing of the manuscript).

Major concerns:
1. Overall meaning of the results. While the data is compelling, at the end of the manuscript the reader is left wondering why this is important in the grand scheme of things. Why does this genetic interaction exist? what is the purpose of engaging these NHRs in response to loss of a single ALH? Perhaps the manuscript would be aided by an enhanced summary figure that puts into context the physiological significance of the work?

*These are good questions. We concede that the strong genetic interaction between alh-4 and nhr-49 requires further analysis beyond the scope of this paper. So far, our understanding of the physiological significance is limited: activation of NHR-49 promotes peroxisome proliferation and function to ensure proper growth and reproduction of ALH-4 deficient worms.*

*Loss of function of alh-4 results in the accumulation of the long chain fatty aldehydes and their derivatives. But the cytotoxicity of long chain fatty aldehydes is not well known even though mutations in aldh3a2, the human ortholog of alh-4, has been known to cause Sjögren-Larsson Syndrome for decades. Without knowing how long fatty aldehydes harm cells, it is hard to know exactly how NHR-49 activation helps to relieve the stress.*

*Recently, we performed a forward genetic screen for mutations that could suppress the synthetic sterility of alh-4(-); nhr-49(lf) worms. Cloning of these new mutants may provide further insights into the genetic interaction between nhr-49 and alh-4 in the future.*

2. Related to #1, the last figure identifies a physiological consequence of the genetic mutations study. The 30% reduction in progeny is a strong piece of data, but I am left wondering if this is a defect in oocyte, sperm, or overall change in reproductive tissue function. The author should test whether mating can rescue this phenotype and/or test if mutant male sperm have a defect in dominating fertilization.

*We set up genetic crosses as suggested. After mating with WT male, alh-4(-) hermaphrodites had a reduced brood size (about 17% reduction) comparing to WT hermaphrodites. Although the difference was not statistically significant, we could not rule out oocyte defects in alh-4(-) worms (Fig. VIIA).*

*In the offspring from the mated hermaphrodites, we noticed that the percentage of males was much lower than 50% after mating with alh-4(-) males (Fig. VIIB). Using the number of the male offspring as an indicator of male fertility, alh-4(-) males showed significant reduction of fertility comparing to WT males (Fig. VIIC). The reduced male fertility could be due to poor sperm quality or defects in mating behavior. The determination of the precise mechanism is beyond the scope of this paper.*

Figure VII. Reduced fertility of *alh-4(-)* worms. (A) Quantification of brood size, (B) quantification of the percentage of male offsprings and (C) quantification of the number of male offsprings. n >= 8 for each group. In the plot, mean ± SEM of each group is shown. For statistical tests, *p < 0.05, **p < 0.01, ***p < 0.001, ****p < 0.0001, the actual p-value is displayed when p is between 0.05 and 0.1, n.s. (not significant) p > 0.1. Two-tailed unpaired Student’s t-test was applied in A&C. Two-way ANOVA with Tukey’s multiple comparisons test was applied in B. Genetic crosses were performed by mixing L4 hermaphrodites with 1-day old adult males in a 1:2 ratio on mating plates for 1 day.

*The germ cell defects of alh-4 mutant worms may be partially due to alh-4 deficiency in somatic cells. This is because the synthetic sterile phenotype of alh-4(-); nhr-49(lf) worms can be rescued by the expression of either nhr-49 in the intestine or the hypodermis, or alh-4 in somatic cells using extra-chromosomal arrays (Fig. VI and Fig. 8A), which cannot direct gene expression in the germline.*

3. Several of the claims in the manuscript are correlative when they could really be tested and shown to be causal.
3a. For example, the authors state "It is plausible that these catalases may protect alh-4(-) worms against exogenous hydrogen peroxide treatment (Fig S4B)." The authors should either test this directly in a ctl mutant background, or move statements like this to the discussion.

*We agree with you and have moved the sentence.*

The change in the manuscript:

The sentence “It is plausible that these catalases may protect alh-4(-) worms against exogenous hydrogen peroxide treatment (Fig S4B).” has been deleted.

3b. Similarly,the use of loss of function and gain of function alleles is a strength, but why was this specific nhr-49 gain of function allele chosen? Work from the Taubert lab suggests that different nhr-49gf alleles have specific activities. This should at a minimum be discussed further.

*Thank you for the suggestion. There are three known nhr-49 gain of function alleles, nhr-49(et7), nhr-49(et8) and nhr-49(et13) (Svensk, Emma, et al. “PAQR-2 Regulates Fatty Acid Desaturation during Cold Adaptation in C. Elegans.” PLoS Genetics, vol. 9, no. 9, 2013, p. e1003801, doi:10.1371/journal.pgen.1003801). Later on, a more comprehensive characterization of each allele was carried out by the Taubert lab. They found that all three alleles enhanced the transcriptional activation function of NHR-49. In addition, nhr-49(et13) removed the transcriptional repression function of NHR-49 (Lee, Kayoung, et al. “Gain-of-Function Alleles in Caenorhabditis Elegans Nuclear Hormone Receptor Nhr-49 Are Functionally Distinct.” PLoS ONE, vol. 11, no. 9, 2016, pp. 1–24, doi:10.1371/journal.pone.0162708.).*

*In order to focus on gene activation by NHR-49 in ALH-4 deficient worms, we could have used nhr-49(et7) or nhr-49(et8). We chose to use nhr-49(et8) because we already had the allele in our lab. Requesting nhr-49(et7) from the CGC would have caused unnecessary delay to our study.*

*We found that worms bearing the nhr-49(et8) allele phenocopied alh-4(-) worms in many aspects: enhanced expression of daf-22, increased peroxisome number and smaller lipid droplets. In our study, the NHR-49 dependent transcriptional response in alh-4 mutant worms was further demonstrated by additional experiments that involved the use of nhr-49 deletion allele, nhr-49 RNAi and the comparison of the gene expression profiles of nhr-49(lf) and alh-4(-) worms.*

The change in the manuscript:

A brief discussion has been added before the presentation of the data about *nhr-49(gf)*/ *nhr-49(et8)*.

“Therefore, NHR-49 and NHR-79 appeared to act in concert to regulate *daf-22* expression. Our conclusion was further substantiated by the following observation: the elevation of *daf-22* expression in *nhr-49* gain-of-function (*gf*) mutant worms was partially dependent on *nhr-79* (Fig 7A-B).”

has been replaced by

“Therefore, NHR-49 and NHR-79 appeared to act in concert to regulate *daf-22* expression. Next, we used an *nhr-49* gain-of-function (*gf*) allele to further substantiate our conclusion. There are three gain of function alleles of *nhr-49*: *nhr-49(et7), nhr-49(et8)* and *nhr-49(et13)* [36]. All alleles enhance the transcriptional activity of NHR-49 while *nhr-49(et13)* also causes de-repression of genes that are normally repressed by NHR-49 [54]. We chose *nhr-49(et8)* for our analysis, in order to focus on gene activation by NHR-49, in response to ALH-4 deficiency. We found that *daf-22* expression in *nhr-49(gf)* worms was elevated and such an effect was partially dependent on *nhr-79* (Fig 7A-B).”

Minor concerns:
Without protein function (biochemical and enzymatic) the authors should limit their interpretations of the data to the genetic interactions.

*Thank you for the suggestion and we have revised the conclusion of the study of ALH-4ΔC and ALH-4ΔGK. Please refer the response under the comment 4 of Reviewer #3.*

The use of active and passive voice (and switching in and out of present/past tense) is awkward and should be edited.

Past tense is applied when describing the experimental results we observed while present tense is applied when we draw conclusion.

*Thank you for your suggestion and we have checked the text and edited those problems.*

The change in the manuscript:

In line 144: “We concluded that the loss of *alh-4* function suppressed LD expansion without compromising LD biogenesis.” has been replaced with “We conclude that the loss of *alh-4* function suppresses LD expansion without compromising LD biogenesis”;

In line 177: “appeared” has been replaced with “appears”;

In line 184: “associated” has been replaced with “associates”;

In line 186: “shared” has been replaced with “share”;

In line 206: “was” has been replaced by “is”;

In line 213:“was” has been replaced by “is”;

In line 234:“suggested” has been replaced by “suggest”;

In line 262:“was” has been replaced by “is”;

In line 295:“corroborated” has been replaced by “corroborate”;

In line 308:“were” has been replaced by “are”;

In line 312:“appeared” has been replaced by “appear”;

In line 317:“suggested” has been replaced by “suggests”;

In line 353:“were” has been replaced by “are”;

In line 359:“appeared” has been replaced by “appear”;
